# Existence of mild solutions for fractional nonautonomous evolution equations of Sobolev type with delay

**DOI:** 10.1186/s13660-017-1526-5

**Published:** 2017-10-10

**Authors:** Haide Gou, Baolin Li

**Affiliations:** College of Mathematics and Statistics, Northwest Normal University, Lanzhou, 730070 P.R. China

**Keywords:** 34K30, 34K45, 35B10, 47D06, evolution equations, mild solutions, Hilfer fractional derivative, noncompact measure

## Abstract

In this paper, we deal with a class of nonlinear fractional nonautonomous evolution equations with delay by using Hilfer fractional derivative, which generalizes the famous Riemann-Liouville fractional derivative. The definition of mild solutions for the studied problem was given based on an operator family generated by the operator pair $(A,B)$ and probability density function. Combining the techniques of fractional calculus, measure of noncompactness, and fixed point theorem with respect to *k*-set-contractive, we obtain a new existence result of mild solutions. The results obtained improve and extend some related conclusions on this topic. At last, we present an application that illustrates the abstract results.

## Introduction

Fractional differential equations have been successfully applied to various fields, for example, physics, engineering, chemistry, aerodynamics, electrodynamics of complex medium, and polymer rheology, and they have been emerging as an important area of investigation in the last few decades; see [1–5]. In [6–12], the authors discussed the existence of solutions for various nonlinear differential equations or partial differential equations by measures of noncompactness and fixed point theorems, whereas in [13–16], the authors investigated the existence of solutions for the evolution equations by the monotone iterative method.

On the other hand, Hilfer [7] proposed a generalized Riemann-Liouville fractional derivative (for short, the Hilfer fractional derivative), which includes the Riemann-Liouville and Caputo fractional derivatives. Furati et al. [17] considered an initial value problem for a class of nonlinear fractional differential equations involving the Hilfer fractional derivative. Very recently, Gu and Trujillo [18] investigated a class of evolution equations involving the Hilfer fractional derivatives by using Mittag-Leffler functions. To the best of our knowledge, there are no results about nonlinear fractional nonautonomous evolution equations of Sobolev type with delay.

Motivated by the above discussion, in this paper, we use the fixed point theorems combined with the theory of propagation family to discuss the existence of mild solutions for nonlinear fractional nonautonomous evolution equations of Sobolev type with delay of the form

1.1 $$ \textstyle\begin{cases} D^{\nu ,\mu }_{0+}Bu(t)=Au(t)+Bf(t,u(\tau_{1}(t)),\ldots ,u(\tau_{m}(t))),& t\in J, \\ I^{(1-\nu )(1-\mu )}_{0+}Bu(0)=Bu_{0}, \end{cases} $$

where $D^{\nu ,\mu }_{0+}$ is the Hilfer fractional derivative, which will be given in next section, $0\leq \nu \leq 1, 0<\mu <1$, the state $u(\cdot )$ takes values in a Banach space *E*, $J=[0,b]$ ($b>0$), $J'=(0,b]$, *A* and *B* are closed (unbounded) linear operators with domains contained in *E*, *m* is a positive integer number, $\tau_{k}:J\rightarrow J$ are continuous functions such that $0\leq \tau_{k}(t)\leq t$ for $k=1,2,\ldots ,m, f:J\times E^{m}\rightarrow E$ is a continuous function, and $u_{0}\in D(B)$.

Furthermore, we study problem (1.1) without assuming that *B* has bounded (or compact) inverse and without any assumption on the relation between $D(A)$ and $D(B)$. Our purpose is to introduce the theory of propagation family $\{W(t)\}_{t\geq 0}$ (an operator family generated by the operator pair $(A,B)$; see Definition 2.7) from Jin Liang and Ti-Jun Xiao [19] and probability density function and then to give a proper definition of mild solutions for nonlinear fractional nonautonomous evolution equations (1.1), which plays a key role in our discussion. The existence of a mild solution for problem (1.1) is obtained under certain assumptions on the nonlinear term *f* by using the Hilfer fractional derivative, measure of noncompactness, and fixed point theorem. At last, as an application, we also obtain the existence of mild solutions for the nonlinear time fractional reaction-diffusion equation

1.2 $$ \textstyle\begin{cases} ^{C}D^{\nu ,\mu }_{t}u(x,t)-a(t)\Delta u(x,t)=f(t,u(x,\tau_{1}(t)),\ldots ,u(x,\tau_{m}(t))),&t\in J, \\ u(x,t)=0,&x\in \partial \Omega ,t\in J, \\ u(x,0)=\varphi (x),&x\in \Omega , \end{cases} $$

introduced by Ouyang [20] and Zhu, Liu, and Wu [21], where Δ is the Laplace operator, $\Omega \in R^{m}$ is a bounded domain with a sufficiently smooth boundary *∂*Ω, $f : J\times R^{m} \rightarrow R $ is a nonlinear function, and $\varphi \in L^{2}( \Omega )$.

The rest of this paper is organized as follows: In Section 2, we recall some basic known results and introduce some notations. In Section 3, we discuss the existence theorems of mild solutions for problem (1.1). At last, two examples are presented to illustrate the main results.

## Preliminaries

In this section, we briefly recall some basic known results. Throughout this work, we set $J = [0, b]$, where $b >0$ is a constant. Let *E* be a Banach space with the norm $\Vert \cdot \Vert $, and let the pair $(A,B)$ generate a propagation family $\{W(t)\}_{t\geq 0}$ (see Definition 2.7). We denote by $B(E)$ the Banach space of all bounded linear operators from *E* to *E* and denote by $C(J,E)$ the Banach space of all continuous *E*-valued functions on the interval *J* with norm $\Vert u \Vert =\max_{t\in J}\Vert u(t) \Vert $. Let

$$ C^{\nu ,\mu }(J,E)= \Bigl\{ u\in C \bigl(J',E \bigr):\lim _{t\rightarrow 0^{+}}t^{(1- \nu )(1-\mu )}u(t) \mbox{ exists and is finite} \Bigr\} $$

with norm $\Vert \cdot \Vert _{\nu ,\mu }$ defined by

$$ \Vert u \Vert _{\nu ,\mu }=\sup_{0\leq t\leq b} \bigl\vert t^{(1-\nu )(1-\mu )}u(t) \bigr\vert . $$

Evidently, $C^{\nu ,\mu }(J,E)$ is a Banach space.

For completeness, we recall the following definitions from fractional calculus.

### Definition 2.1

The Riemann-Liouville fractional integral of order *α* of a function $f:[0,\infty )\rightarrow R$ is defined as

$$ I_{0+}^{\alpha }f(t)=\frac{1}{\Gamma (\alpha )} \int_{0}^{t}(t-s)^{ \alpha -1}f(s)\,ds,\quad t>0,\alpha>0, $$

provided that the right-hand side is pointwise defined on $(0,\infty )$.

### Definition 2.2

The Riemann-Liouville derivative of order *α* with the lower limit zero for a function $f:[0,\infty )\rightarrow R$ can be written as

$$ D^{\alpha }_{0^{+}}f(t)=\frac{1}{\Gamma (n-\alpha )}\frac{d^{n}}{dt ^{n}} \int_{0}^{t}\frac{f(s)}{(t-s)^{\alpha +1-n}}\,ds, \quad t>0,n-1< \alpha < n. $$

### Definition 2.3

The Caputo fractional derivative of order *α* for a function $f:[0,\infty )\rightarrow R$ can be written as

$$ ^{c}D^{\alpha }_{0^{+}}f(t)=D^{\alpha }_{0^{+}} \Biggl[f(t)-\sum_{k=0} ^{n-1} \frac{t^{k}}{k!}f^{(k)}(0) \Biggr],\quad t>0,n-1< \alpha < n, $$

where $n = [\alpha ] + 1$, and $[\alpha ]$ denotes the integer part of *α*.

If *u* is an abstract function with values in *E*, then the integrals appearing in Definitions 2.2 and 2.3 are taken in Bochner’s sense.

### Definition 2.4

Hilfer fractional derivative; see [7]

The generalized Riemann-Liouville fractional derivative of order $0\leq \nu \leq 1$ and $0<\mu <1$ with lower limit *a* is defined as

$$ D^{\nu ,\mu }_{a+}f(t)=I^{\nu (1-\mu )}_{a+} \frac{d}{dt}I^{(1-\nu )(1- \mu )}_{a+}f(t) $$

for functions such that the expression on the right-hand side exists.

Recently (Hilfer et al. [22]), this definition for $n-1 < \mu \leq n, n \in N, 0 \leq \nu \leq 1$, was rewritten in a more general form:

$$\begin{aligned} D^{\nu ,\mu }_{a+}f(t) &=I^{\nu (n-\mu )}_{a+} \frac{d^{n}}{dt^{n}}I^{(1- \nu )(n-\mu )}_{a+}f(t) \\ &=I^{\nu (n-\mu )}_{a+}D_{a+}^{\mu +\nu n- \mu \nu }f(t), \end{aligned}$$

where $D_{a+}^{\mu +\nu n-\mu \nu }$ is the Riemann-Liouville fractional derivative, and $I_{a+}^{\nu (n-\mu )}$ is the Riemann-Liouville integral.

### Remark 2.1

(i) When $\nu =0,0<\mu <1$, and $a=0$, the Hilfer fractional derivative corresponds to the classical Riemann-Liouville fractional derivative:
  $$ D_{0+}^{0,\mu }f(t)=\frac{d}{dt}I^{1-\mu }_{0+}f(t)=D^{\mu }_{0+}f(t). $$
(ii) When $\nu =1, 0<\mu <1$, and $a=0$, the Hilfer fractional derivative corresponds to the classical Caputo fractional derivative:
  $$ D^{1,\mu }_{0+}f(t)=I^{1-\mu }_{0+} \frac{d}{dt}f(t)={}^{c}D^{\mu }_{0+}f(t). $$

Now, we recall the basic definitions and properties of the Kuratowski measure of noncompactness.

### Definition 2.5

[23]

Let *E* be a Banach space, and let $\Omega_{E}$ be the bounded subsets of *E*. The Kuratowski measure of noncompactness is the map $\alpha :\Omega_{E}\rightarrow [0,\infty )$ defined by (here $B\in \Omega_{E}$)

$$ \alpha (B)=\inf \Biggl\{ \varepsilon >0: B=\bigcup_{i=1}^{n} B_{i} \mbox{ and }\operatorname{diam}(B_{i})\leq \varepsilon \mbox{ for }i=1,\ldots ,n \Biggr\} , $$

where $\operatorname{diam}B_{i}=\sup \{\vert x-y \vert :x,y\in B_{i}\}$.

### Lemma 2.1

[24]

*Let* *S* *and* *T* *be bounded sets of* *E*, *and let* *a* *be a real number*. *Then the noncompactness measure has the following properties*:

1. $\alpha (S)=0$ *if and only if S is a relatively compact set*.
2. $S\subset T$ *implies that* $\alpha (S)\leq \alpha (T)$.
3. $\alpha (\overline{S}) = \alpha (S)$.
4. $\alpha (S\cup T) =\max \{\alpha (S),\alpha (T)\}$.
5. $\alpha (aS) = \vert a \vert \alpha (S)$.
6. $\alpha (S + T) \leq \alpha (S) +\alpha (T)$.
7. $\alpha (\overline{{\operatorname{co}}}S) = \alpha (S)$, *where* $\overline{{\operatorname{co}}}S$ *is the convex closure of S*.
8. $\vert \alpha (S)-\alpha (T) \vert \leq 2d_{h}(S,T)$, *where* $d_{h}(S,T)$ *denotes the Hausdorff distance between the sets* *S* *and* *T*, *that is*,
  $$ d_{h}(S,T)=\max \Bigl\{ \sup_{x\in S}d(x,T),\sup _{x\in T}d(x,S) \Bigr\} , $$
  *where* $d(\cdot ,\cdot )$ *denotes the distance from an element of* *E* *to a set of E*.

### Lemma 2.2

[25]

*Let* *E* *be a Banach space*, *and let* $D\subset E$ *be bounded*. *Then there exists a countable set* $D_{0} \subset D$ *such that* $\alpha (D) \leq 2\alpha (D_{0})$.

### Lemma 2.3

[26]

*Let* *E* *be a Banach space*, *and let* $\Omega \subset C(J,E)$ *be equicontinuous and bounded*. *Then* $\alpha (\Omega (t))$ *is continuous on* *J*, *and* $\alpha (\Omega )=\max_{t\in J} \alpha (\Omega (t))$.

### Lemma 2.4

[27]

*Let* $\Omega =\{u_{n}\}_{n=1}^{\infty }\subset C(J,E)$ *be a bounded and countable set*. *Suppose that there exists a function* $m\in L^{1}(J,R^{+})$ *such that*, *for every* $n\in N$,

$$ \bigl\Vert u_{n}(t) \bigr\Vert \leq m(t), \quad \textit{a.e. } t\in J. $$

*Then* $\alpha (\Omega (t))$ *is the Lebesgue integral on* *J*, *and*

$$ \alpha \biggl( \biggl\{ \int_{J} u_{n}(t)\,dt: n\in \mathbb{N} \biggr\} \biggr) \leq 2 \int_{J} \alpha \bigl(\Omega (t) \bigr)\,dt. $$

### Definition 2.6

[28]

Let *E* be a Banach space, and let *S* be a nonempty subset of *E*. A continuous mapping $Q:S\rightarrow E$ is said to be *k*-set contractive if there exists a constant $k\in [0,1)$ such that, for every bounded set $D\subset S$,

$$ \alpha \bigl(Q(D) \bigr)\leq k\alpha (D). $$

### Lemma 2.5

[28]

*Let* *E* *be a Banach space*. *Assume that* $D\subset E$ *is a bounded closed convex set on* *E* *and that the operator* $Q:D\rightarrow D$ *is* *k*-*set*-*contractive*. *Then* *Q* *has at least one fixed point in D*.

We recall the abstract degenerate Cauchy problem [14]:

2.1 $$ \textstyle\begin{cases} \frac{d}{dt}Bu(t)=Au(t),& t\in J, \\ Bu(0)=Bu_{0}. \end{cases} $$

### Definition 2.7

See [19], Definition 1.4

A strongly continuous operator family $\{W(t)\}_{t\geq 0}$ of $D(B)$ to a Banach space *E* such that $\{W(t)\}_{t\geq 0}$ is exponentially bounded, which means that, for any $u\in D(B)$, there exist $a>0$ and $M>0$ such that

$$ \bigl\Vert W(t)u \bigr\Vert \leq Me^{at}\Vert u \Vert ,\quad t \geq 0, $$

is called an exponentially bounded propagation family for (2.1) if for $\lambda >a$,

2.2 $$ (\lambda B-A)^{-1}Bu= \int_{0}^{\infty }e^{-\lambda t}W(t)u\,dt,\quad u\in D(B). $$

In this case, we also say that (2.1) has an exponentially bounded propagation family $\{W(t)\}_{t\geq 0}$.

Moreover, if (2.2) holds, we also say that the pair $(A,B)$ generates an exponentially bounded propagation family $\{W(t)\}_{t\geq 0}$.

### Lemma 2.6

[29]

*Problem* (1.1) *is equivalent to the integral equation*

2.3 $$\begin{aligned} Bu(t) &=\frac{Bu_{0}}{\Gamma (\nu (1-\mu )+\mu )}t^{(\nu -1)(1-\mu )} \\ &\quad {}+\frac{1}{\Gamma (\mu )} \int_{0}^{t}(t-s)^{\mu -1} \bigl[Au+Bf \bigl(s,u \bigl(\tau_{1}(s) \bigr), \ldots ,u \bigl( \tau_{m}(s) \bigr) \bigr) \bigr]\,ds,\quad t\in J. \end{aligned}$$

### Lemma 2.7

*If integral* (2.3) *holds*, *then we have*

2.4 $$ u(t)=S_{\nu ,\mu }(t)u_{0}+ \int_{0}^{t}K_{\mu }(t-s)f \bigl(s,u \bigl( \tau_{1}(s) \bigr), \ldots ,u \bigl(\tau_{m}(s) \bigr) \bigr)\,ds, $$

*where*

$$ S_{\nu ,\mu }(t)=I_{0+}^{\nu (1-\mu )}K_{\mu }(t), \quad K_{\mu }(t)= \mu \int_{0}^{\infty }\sigma t^{\mu -1} \xi_{\mu }(\sigma )W \bigl(t^{\mu } \sigma \bigr)u_{0}\,d\sigma . $$

### Proof

Let $\lambda >0$. Applying the Laplace transform

$$\begin{aligned}& \widehat{u}(\lambda )= \int_{0}^{\infty }e^{-\lambda t}u(t)\,dt, \\& v( \lambda )= \int_{0}^{\infty }e^{-\lambda t}f \bigl(t,u \bigl( \tau_{1}(t) \bigr), \ldots ,u \bigl( \tau_{m}(t) \bigr) \bigr)\,dt \end{aligned}$$

to (2.3), we have

$$\begin{aligned} B\widehat{u}(\lambda )=\lambda^{(1-\nu )(1-\mu )-1}Bu_{0}+ \frac{1}{ \lambda^{\mu }}A\widehat{u}(\lambda )+\frac{1}{\lambda^{\mu }}Bv( \lambda ). \end{aligned}$$

Then

$$ \bigl(\lambda^{\mu }B-A \bigr)\widehat{u}(\lambda )= \lambda^{\nu (\mu -1)}Bu_{0}+Bv( \lambda ), $$

and thus

2.5 $$\begin{aligned} \widehat{u}(\lambda ) &=\lambda^{\nu (\mu -1)} \bigl(\lambda^{\mu }B-A \bigr)^{-1}Bu _{0}+ \bigl(\lambda^{\mu }B-A \bigr)^{-1}Bv(\lambda ) \\ &=\lambda^{\nu (\mu -1)} \int_{0}^{\infty }e^{-(\lambda^{\mu }) s}W(s)u _{0}\,ds+ \int_{0}^{\infty }e^{-(\lambda^{\mu }) s}W(s)v(\lambda )\,ds, \end{aligned}$$

provided that the integral in (2.5) exists, where *I* is the identity operator on *E*.

We consider the following one-sided stable probability density in [30]:

$$ \varpi_{\mu }(\sigma )=\frac{1}{\pi }\sum _{n=1}^{\infty }(-1)^{n-1} \sigma^{-\mu n-1} \frac{\Gamma (n\mu +1)}{n!}\sin (n\pi \mu ),\quad \sigma \in (0,\infty ), $$

whose Laplace transform is given by

2.6 $$ \int_{0}^{\infty }e^{-\lambda \sigma }\varpi_{\mu }( \sigma )\,d\sigma =e^{-\lambda^{\mu }},\quad \mu \in (0,1). $$

Then, by $s=t^{\mu }$ and (2.6) we have

2.7 $$\begin{aligned} &\int_{0}^{\infty }e^{-\lambda^{\mu }s}W(s)u_{0}\,ds \\ &\quad = \int_{0}^{\infty} \mu t^{\mu -1}e^{-(\lambda t)^{\mu }}W \bigl(t^{\mu } \bigr)u_{0}\,dt \\ &\quad = \int_{0}^{\infty } \int_{0}^{\infty }e^{-(\lambda t \sigma )}\mu t ^{\mu -1} \varpi_{\mu }(\sigma )W \bigl(t^{\mu } \bigr)u_{0}\,d\sigma \,dt \\ &\quad =\mu \int_{0}^{\infty } \int_{0}^{\infty }e^{-\lambda \theta }\frac{ \theta^{\mu -1}}{\sigma^{\mu }} \varpi_{\mu }(\sigma )W \biggl(\frac{ \theta^{\mu }}{\sigma^{\mu }} \biggr)u_{0}\,d\theta \,d\sigma \\ &\quad = \int_{0}^{\infty }e^{-\lambda \tau } \biggl[\mu \int_{0}^{\infty }\frac{ \tau^{\mu -1}}{\sigma^{\mu }}\varpi_{\mu }( \sigma )W \biggl(\frac{\tau^{ \mu }}{\sigma^{\mu }} \biggr)u_{0}\,d\sigma \biggr]\,d\tau \\ &\quad = \int_{0}^{\infty }e^{-\lambda t} \biggl[\mu \int_{0}^{\infty }\frac{t ^{\mu -1}}{\sigma^{\mu }}\varpi_{\mu }( \sigma )W \biggl(\frac{t^{\mu }}{ \sigma^{\mu }} \biggr)u_{0}\,d\sigma \biggr]\,dt. \end{aligned}$$

2.8 $$\begin{aligned} &\int_{0}^{\infty } e^{-\lambda^{\mu }s}W(s)v(\lambda )\,ds \\ &\quad = \int_{0} ^{\infty }\mu t^{\mu -1}e^{-(\lambda t)^{\mu }} W \bigl(t^{\mu } \bigr) \biggl( \int_{0} ^{\infty }e^{-\lambda s}f \bigl(s,u \bigl( \tau_{1}(s) \bigr),\ldots ,u \bigl(\tau_{m}(s) \bigr) \bigr)\,ds \biggr)\,dt \\ &\quad = \int_{0}^{\infty } \int_{0}^{\infty }e^{-(\lambda t \sigma )}\mu t ^{\mu -1} \varpi_{\mu }(\sigma ) \\ &\quad \quad {} \times W \bigl(t^{\mu } \bigr) \biggl( \int_{0}^{\infty }e ^{-\lambda s}f \bigl(s,u \bigl( \tau_{1}(s) \bigr),\ldots ,u \bigl(\tau_{m}(s) \bigr) \bigr)\,ds \biggr)\,d\sigma \,dt \\ &\quad =\mu \int_{0}^{\infty } \int_{0}^{\infty }e^{-\lambda \theta }\frac{ \theta^{\mu -1}}{\sigma^{\mu }} \varpi_{\mu }(\sigma ) \\ &\quad\quad {}\times W \biggl(\frac{ \theta^{\mu }}{\sigma^{\mu }} \biggr) \biggl( \int_{0}^{\infty }e^{-\lambda s}f \bigl(s,u \bigl( \tau_{1}(s) \bigr),\ldots ,u \bigl(\tau_{m}(s) \bigr) \bigr)\,ds \biggr)\,d\theta \,d\sigma \\ &\quad =\mu \int_{0}^{\infty } \biggl( \int_{0}^{\infty } \int_{s}^{\infty }e ^{-\lambda t}\frac{(t-s)^{\mu -1}}{\sigma^{\mu }} \varpi_{\mu }(\sigma) \\ &\quad \quad {}\times W \biggl(\frac{(t-s)^{\mu }}{\sigma^{\mu }} \biggr)f \bigl(s,u \bigl( \tau_{1}(s) \bigr), \ldots ,u \bigl(\tau_{m}(s) \bigr) \bigr)\,dt \,ds \biggr) \,d\sigma \\ &\quad =\mu \int_{0}^{\infty } \biggl( \int_{0}^{\infty } \int_{0}^{t} e^{- \lambda t}\frac{(t-s)^{\mu -1}}{\sigma^{\mu }} \varpi_{\mu }(\sigma) \\ &\quad \quad {}\times W \biggl(\frac{(t-s)^{\mu }}{\sigma^{\mu }} \biggr)f \bigl(s,u \bigl( \tau_{1}(s) \bigr),\ldots ,u \bigl(\tau_{m}(s) \bigr) \bigr)\,ds \,dt \biggr) \,d\sigma \\ &\quad = \int_{0}^{\infty }e^{-\lambda t} \biggl[\mu \int_{0}^{t} \int_{0}^{ \infty }\frac{(t-s)^{\mu -1}}{\sigma^{\mu }}\varpi_{\mu }( \sigma ) \\ &\quad \quad {}\times W \biggl(\frac{(t-s)^{\mu }}{\sigma^{\mu }} \biggr)f \bigl(s,u \bigl( \tau_{1}(s) \bigr),\ldots ,u \bigl(\tau_{m}(s) \bigr) \bigr)\,d\sigma ds \biggr]\,dt. \end{aligned}$$

Thus, it follows from (2.5), (2.7), and (2.8) that, for $t\in J$,

$$\begin{aligned} \widehat{u}(\lambda ) &=\lambda^{\nu (\mu -1)} \int_{0}^{\infty }e^{- \lambda t} \biggl[\mu \int_{0}^{\infty }\frac{t^{\mu -1}}{\sigma^{\mu }} \varpi_{\mu }(\sigma )W \biggl(\frac{t^{\mu }}{\sigma^{\mu }} \biggr)u_{0}\,d\sigma \biggr]\,dt \\ &\quad {}+ \int_{0}^{\infty }e^{-\lambda t} \biggl[\mu \int_{0}^{t} \int_{0}^{ \infty }\frac{(t-s)^{\mu -1}}{\sigma^{\mu }}\varpi_{\mu }( \sigma ) \\ &\quad {}\times W \biggl(\frac{(t-s)^{\mu }}{\sigma^{\mu }} \biggr)f \bigl(s,u \bigl( \tau_{1}(s) \bigr),\ldots ,u \bigl(\tau_{m}(s) \bigr) \bigr)\,d\sigma ds \biggr]\,dt. \end{aligned}$$

Since the Laplace inverse transform of $\lambda^{\nu (\mu -1)}$ is

$$ \mathcal{L}^{-1} \bigl(\lambda^{\nu (\mu -1)} \bigr)= \textstyle\begin{cases} \frac{t^{\nu (1-\mu )-1}}{\Gamma (\nu (1-\mu ))},&0< \nu < 1, \\ \delta (t),&\nu =0, \end{cases} $$

where $\delta (t)$ is the delta function, we invert the last Laplace transform to obtain

2.9 $$\begin{aligned} u(t) &= \biggl(\mathcal{L}^{-1} \bigl(\lambda^{\nu (\mu -1)} \bigr)\times \biggl( \int_{0} ^{\infty }\mu \frac{t^{\mu -1}}{\sigma^{\mu }} \varpi_{\mu }(\sigma ) W \biggl(\frac{t^{\mu }}{\sigma^{\mu }} \biggr)\,d\sigma \biggr) (t) \biggr)u_{0} \\ &\quad {}+\mu \int_{0}^{t} \int_{0}^{\infty }\frac{(t-s)^{\mu -1}}{\sigma^{ \mu }}\varpi_{\mu }( \sigma ) W \biggl(\frac{(t-s)^{\mu }}{\sigma^{\mu }} \biggr)f \bigl(s,u \bigl( \tau_{1}(s) \bigr),\ldots ,u \bigl(\tau_{m}(s) \bigr) \bigr)\,d\sigma \,ds \\ &= \int_{0}^{t}\frac{t^{\nu (1-\mu )-1}}{\Gamma (\nu (1-\mu ))} \int_{0} ^{\infty }\mu \sigma t^{\mu -1} \xi_{\mu }(\sigma )W \bigl(t^{\mu } \sigma \bigr)u _{0}\,d\sigma \\ &\quad {}+\mu \int_{0}^{t} \int_{0}^{\infty }\sigma (t-s)^{\mu -1} \xi_{\mu }( \sigma ) \\ &\quad {} \times W \bigl((t-s)^{\mu }\sigma \bigr)f \bigl(s,u \bigl(\tau_{1}(s) \bigr),\ldots ,u \bigl(\tau_{m}(s) \bigr) \bigr)\,d\sigma \,ds \\ &= \bigl(I^{\nu (1-\mu )}_{0+}K_{\mu }(t) \bigr)u_{0}+ \int_{0}^{t}K_{ \mu }(t-s)f \bigl(s,u \bigl( \tau_{1}(s) \bigr),\ldots ,u \bigl(\tau_{m}(s) \bigr) \bigr)\,ds \\ &=S_{\nu ,\mu }(t)u_{0}+ \int_{0}^{t}K_{\mu }(t-s)f \bigl(s,u \bigl( \tau_{1}(s) \bigr), \ldots ,u \bigl(\tau_{m}(s) \bigr) \bigr)\,ds, \end{aligned}$$

where $\xi_{\mu }$ is the probability density function defined on $(0,\infty )$ by

$$ \xi_{\mu }(\sigma )=\frac{1}{\mu }\sigma^{-1-\frac{1}{\mu }} \varpi_{ \mu } \bigl(\sigma^{-\frac{1}{\mu }} \bigr)\geq 0. $$

This completes the proof. □

Based on Lemma 2.7, we give the following definition of a mild solution of problem (1.1).

### Definition 2.8

By a mild solution of problem (1.1) we mean a function $u\in C(J',E) $ that satisfies

2.10 $$ u(t)=S_{\nu ,\mu }(t)u_{0}+ \int_{0}^{t}K_{\mu }(t-s)f \bigl(s,u \bigl( \tau_{1}(s) \bigr), \ldots ,u \bigl(\tau_{m}(s) \bigr) \bigr)\,ds, \quad t\in J'. $$

### Remark 2.2

(i) By (2.9) it is easy to see that
  $$ D^{\nu (1-\mu )}_{0+}S_{\nu ,\mu }(t)=K_{\mu }(t),\quad t\in J'. $$
(ii) When $\nu =0$, the fractional equation (1.1) simplifies to the classical Riemann- Liouville fractional equation studied by Zhou et al. [31]. In this case,
  $$ S_{0,\mu }(t)=K_{\mu }(t), \quad t\in J'. $$
(iii) When $\nu =1$, the fractional equation (1.1) simplifies to the classical Caputo fractional equation studied by Zhou and Jiao [32]. In this case,
  $$ S_{1,\mu }(t)=S_{\mu }(t), \quad t\in J, $$
  where $S_{\mu }(t)$ is defined in [32].

### Lemma 2.8

*Assume that* $\{W(t)\}_{t\geq 0}$ *is a norm*-*continuous family for* $t>0$, $\Vert W(t) \Vert \leq M$ *for any fixed* $t>0$, $\{K_{\mu }(t)\}_{t>0}$, *and* $\{S_{\nu ,\mu }(t)\}_{t>0}$ *are linear operators*, *and for any* $u\in E$,

$$ \bigl\Vert K_{\mu }(t) \bigr\Vert \leq \frac{Mt^{\mu -1}}{\Gamma (\mu )},\qquad \bigl\Vert S_{\nu ,\mu }(t) \bigr\Vert \leq \frac{Mt^{(\nu -1)(\mu -1)}}{\Gamma (\nu (1- \mu )+ \mu )}. $$

### Proof

Noting that $\int_{0}^{\infty }\xi_{\mu }(\sigma )\,d\sigma =1$, we get

$$ \biggl\Vert \int_{0}^{\infty }\xi_{\mu }(\sigma )W \bigl(t^{\mu }\sigma \bigr)\,d\sigma \biggr\Vert \leq M. $$

By [33] direct calculation gives that

$$ \int_{0}^{\infty }\sigma \xi_{\mu }(\sigma )\,d\sigma = \int_{0}^{\infty }\frac{1}{\sigma^{\mu }}\varpi_{\mu }( \sigma )\,d\sigma =\frac{1}{ \Gamma (1+\mu )}. $$

Hence, we have

$$ \bigl\Vert K_{\mu }(t) \bigr\Vert \leq \frac{Mt^{\mu -1}}{\Gamma (\mu )}, \quad t>0. $$

For $t\in J'$ and $u\in E$, we have

$$\begin{aligned} \bigl\Vert S_{\nu ,\mu }(t)u \bigr\Vert &= \bigl\Vert I_{0+}^{\nu (1-\mu )}K_{\mu }(t)u \bigr\Vert \\ &= \biggl\Vert \frac{1}{ \Gamma (\nu (1-\mu ))} \int_{0}^{t}(t-s)^{\nu (1-\mu )-1}K_{\mu }(s)u\,ds \biggr\Vert \\ &= \biggl\Vert \frac{t^{(\nu -1)(1-\mu )}}{\Gamma (\nu (1-\mu ))} \int_{0}^{1}(1-s)^{\nu (1-\mu )-1}K_{\mu }(s)u\,ds \biggr\Vert \\ &\leq \frac{t^{(\nu -1)(1-\mu )}M}{\Gamma (\nu (1-\mu ))\Gamma ( \mu )} \int_{0}^{1}(1-s)^{\nu (1-\mu )-1}s^{\mu -1}\,ds \Vert u \Vert \\ &=\frac{Mt^{(\nu -1)(\mu -1)}}{\Gamma (\nu (1-\mu )+\mu )}\Vert u \Vert . \end{aligned}$$

This completes the proof. □

### Lemma 2.9

*Assume that* $\{W(t)\}_{t\geq 0}$ *is a norm*-*continuous family for* $t>0$, $\Vert W(t) \Vert \leq M$, *and* $\{K_{\mu }(t)\}_{t>0}$ *and* $\{S_{\nu ,\mu }(t) \}_{t>0}$ *are strongly continuous for* $t>0$.

### Proof

For any $u\in E$ and $0< t_{1}<t_{2}\leq b$, we have

$$\begin{aligned} & \bigl\Vert K_{\mu }(t_{2})u-K_{\mu }(t_{1})u \bigr\Vert \\ &\quad \leq \biggl\Vert \int_{0}^{\infty }\mu \sigma \xi_{\mu }(\sigma ) \bigl[t_{2}^{\mu -1}W \bigl(t_{2}^{\mu }\sigma \bigr)-t_{1}^{\mu -1}W \bigl(t_{1}^{\mu } \sigma \bigr) \bigr]u\,d\sigma \biggr\Vert \\ &\quad \leq \int_{0}^{\infty }\mu \sigma \xi_{\mu }(\sigma )\,d\sigma \bigl[t _{2}^{\mu -1} \bigl\Vert W \bigl(t_{2}^{\mu } \theta \bigr)-W \bigl(t_{1}^{\mu }\theta \bigr) \bigr\Vert + \bigl\Vert t_{2}^{\mu -1}-t_{1}^{\mu -1} \bigl\Vert W \bigl(t_{1}^{\mu }\sigma \bigr) \bigr]\cdot \bigr\Vert u \bigr\Vert \\ &\quad \leq \frac{1}{\Gamma (\mu )} \bigl[t_{2}^{\mu -1} \bigl\Vert W \bigl(t_{2}^{\mu }\theta \bigr)-W \bigl(t_{1}^{\mu }\theta \bigr)\bigr\Vert + \bigl\Vert t_{2}^{\mu -1}-t_{1}^{\mu -1}\bigr\Vert W \bigl(t _{1}^{\mu }\sigma \bigr) \bigr]\cdot \Vert u \Vert . \end{aligned}$$

Since $W(t)$ is a norm-continuous family for $t>0$, we have

$$ \bigl\Vert K_{\mu }(t_{2})u-K_{\mu }(t_{1})u \bigr\Vert \rightarrow 0\quad \mbox{as } t_{2} \rightarrow t_{1}. $$

For $u\in E$ and $0< t_{1}< t_{2}\leq b$, we get

$$\begin{aligned} & \bigl\Vert S_{\nu ,\mu }(t_{2})u-S_{\nu ,\mu }(t_{1})u \bigr\Vert \\ &\quad =\frac{1}{\Gamma ( \nu (1-\mu ))} \biggl\Vert \int_{0}^{t_{2}}(t_{2}-s)^{\nu (1-\mu )-1}K_{\mu }(s)u \,ds- \int_{0}^{t_{1}}(t_{1}-s)^{\nu (1-\mu )-1}K_{\mu }(s)u\,ds \biggr\Vert \\ &\quad \leq \frac{1}{\Gamma (\nu (1-\mu ))} \biggl\Vert \int_{t_{1}}^{t_{2}}(t_{2}-s)^{\nu (1-\mu )-1}K_{\mu }(s)u\,ds \biggr\Vert \\ &\quad \quad {}+\frac{1}{\Gamma (\nu (1-\mu ))} \biggl\Vert \int_{0}^{t_{1}} \bigl((t_{2}-s)^{\nu (1-\mu )-1}-(t_{1}-s)^{\nu (1-\mu )-1} \bigr)K_{\mu }(s)u\,ds \biggr\Vert \\ &\quad \leq \frac{Mt_{1}^{\mu -1}}{\Gamma (\nu (1-\mu ))\Gamma (\mu )}\frac{1}{ \nu (1-\mu )}(t_{2}-t_{1})^{\nu (1-\mu )} \Vert u \Vert \\ &\quad \quad {}+\frac{M}{\Gamma (\nu (1-\mu ))\Gamma (\mu )} \biggl\Vert \int_{0}^{t_{1}} \bigl((t_{2}-s)^{\nu (1-\mu )-1}-(t_{1}-s)^{\nu (1-\mu )-1} \bigr)\,ds \biggr\Vert \Vert u \Vert . \end{aligned}$$

Consequently, we have

$$ \bigl\Vert S_{\nu ,\mu }(t_{2})u-S_{\nu ,\mu }(t_{1})u \bigr\Vert \rightarrow 0\quad \mbox{as } t_{2}\rightarrow t_{1}, $$

that is, $\{S_{\nu ,\mu }\}_{t>0}$ is strongly continuous. This completes the proof. □

## Main results

In this section, we will state and prove our main results. First of all, we introduce the following assumptions:

(H1): $\{W(t)\}_{t\geq 0}$ is a norm-continuous family for $t>0$ and uniformly bounded, that is, there exists $M>1$ such that $\Vert W(t) \Vert \leq M$.
(H2): For some $r>0$, there exist a constant $\rho >0$ and functions $h_{r}\in L^{p}(J,R^{+})$ ($p>\frac{1}{\mu }>1$) such that, for any $t\in J$ and $u_{k}\in E$ satisfying $\Vert u_{k} \Vert \leq r$ for $k=1,2,\ldots ,m$,
  $$\begin{aligned}& \bigl\Vert f(t,u_{1},u_{2},\ldots ,u_{m}) \bigr\Vert \leq h_{t}(t),\qquad \lim_{r\rightarrow +\infty }\inf \frac{\Vert h_{r} \Vert _{L^{p}}}{r}=\rho < + \infty , \\& I^{\mu }_{0+}h_{r}\in C \bigl(J',R^{+} \bigr),\quad \mbox{and}\quad \lim_{t\rightarrow 0+}t^{(1-\nu )(1-\mu )}I^{\mu }_{0+}h_{r}(t)=0. \end{aligned}$$
(H3): There exist positive constant $L_{k}$ ($k=1,2,\ldots ,m$) such that, for any bounded equicontinuous and countable sets $D_{k}\subset E$ ($k=1,2,\ldots ,m$),
  $$ \alpha \bigl(f(t,D_{1},D_{2},\ldots ,D_{m}) \bigr) \leq \sum_{k=1}^{m}L_{k} \alpha (D_{k}),\quad t\in J. $$

### Theorem 3.1

*Assume that the nonlinear function* $f:J\times E^{m}\rightarrow E$ *is continuous and satisfies assumptions* (H1)-(H3). *Then problem* (1.1) *has at least one mild solution in* $C^{\nu ,\mu }(J,E)$, *provided that*

3.1 $$ \frac{M\rho t^{(1-\nu )(1-\mu )}}{\Gamma (\mu )} l_{p,\mu }b^{\mu -\frac{1}{p}}< 1 $$

*and*

$$ \frac{Mb^{\mu }t^{(1-\nu )(1-\mu )}}{\Gamma (1+\mu )}\sum_{k=1}^{m}L _{k}< \frac{1}{4}. $$

### Proof

We consider the operator $Q:C^{\nu ,\mu }(J,E)\rightarrow C^{\nu ,\mu }(J,E)$ defined by

3.2 $$ (Qu) (t)=S_{\nu ,\mu }(t)u_{0}+ \int_{0}^{t}K_{\mu }(t-s)f \bigl(s,u \bigl( \tau_{1}(s) \bigr), \ldots ,u \bigl(\tau_{m}(s) \bigr) \bigr)\,ds, \quad t\in J'. $$

By direct calculation we know that the operator *Q* is well-defined. From Definition 2.8 it is easy to verify that the mild solution of problem (1.1) is equivalent to the fixed point of the operator *Q* defined by (3.2). In the following, we will prove that the operator $Q:C^{\nu ,\mu }(J,E)\rightarrow C^{\nu ,\mu }(J,E)$ has at least one fixed point by applying the fixed point theorem with respect to a *k*-set-contractive operator. Our proof will be divided into four steps.

Let $B_{r}:=\{u\in C^{\nu ,\mu }(J,E):\Vert u(t) \Vert _{\nu ,\mu }\leq r, t \in J\} $. Then $B_{r}$ is a closed and convex subset of $C^{\nu , \mu }(J,E)$. Observe that, for all $u\in B_{r}$,

$$\begin{aligned} &\lim_{t\rightarrow 0+} t^{(1-\nu )(1-\mu )}S_{\nu ,\mu }(t)u_{0} \\ &\quad =\lim_{t\rightarrow 0+}\frac{t^{(1-\nu )(1-\mu )}}{\Gamma (\nu (1- \mu ))} \int_{0}^{t}(t-s)^{\nu (1-\mu )-1}K_{\mu }(s)u_{0}\,ds \\ &\quad =\lim_{t\rightarrow 0+}\frac{1}{\Gamma (\nu (1-\mu ))} \int_{0}^{1}(1-s)^{ \nu (1-\mu )-1}K_{\mu }(s)u_{0}\,ds \\ &\quad =\lim_{t\rightarrow 0+}\frac{1}{\Gamma (\nu (1-\mu ))\Gamma (\mu )} \int_{0}^{1}(1-s)^{\nu (1-\mu )-1}s^{\mu -1}u_{0}\,ds \\ &\quad =\frac{u_{0}}{\Gamma (\nu (1-\mu )+\mu )}. \end{aligned}$$

Define $t^{(1-\nu )(1-\mu )}(Qu)(t)$ as follows:

$$ t^{(1-\nu )(1-\mu )}(Qu) (t):= \textstyle\begin{cases} t^{(1-\nu )(1-\mu )}S_{\nu ,\mu }(t)u_{0} \\ \quad {}+t^{(1-\nu )(1-\mu )}\int_{0}^{t}K_{\mu }(t-s)f(s,u(\tau_{1}(s)), \ldots ,u(\tau_{m}(s)))\,ds,& t\in J', \\ \frac{u_{0}}{\Gamma (\nu (1-\mu )+\mu )}, & t=0. \end{cases} $$

Step 1. We show that there exists $r>0$ such that $QB_{r}\subset B _{r}$. Suppose this is not true. Then for each $r>0$, there exists $u_{r}(\cdot )\in B_{r}$ such that $\Vert (Qu_{r})(t) \Vert >r$ for some $t\in J$. Combining Lemma 2.8, assumptions (H1) and (H2), and the Hölder inequality, we get that

3.3 $$\begin{aligned} r &< \bigl\Vert t^{(1-\nu )(1-\mu )}(Qu_{r}) (t) \bigr\Vert \\ &\leq \bigl\Vert t^{(1-\nu )(1-\mu )}S_{\nu ,\mu }(t)u_{0} \bigr\Vert \\ &\quad {}+ \biggl\Vert t^{(1-\nu )(1-\mu )} \int_{0}^{t}K_{\mu }(t-s)f \bigl(s,u \bigl( \tau_{1}(s) \bigr),\ldots ,u \bigl(\tau_{m}(s) \bigr) \bigr)\,ds \biggr\Vert \\ &\leq \frac{M\Vert u_{0} \Vert }{\Gamma (\nu (1-\mu )+\mu )}+\frac{Mt^{(1- \nu )(1-\mu )}}{\Gamma (\mu )} \int_{0}^{t}(t-s)^{\mu -1}h_{r}(s)\,ds \\ &\leq \frac{M\Vert u_{0} \Vert }{\Gamma (\nu (1-\mu )+\mu )}+\frac{Mt^{(1- \nu )(1-\mu )}}{\Gamma (\mu )} \biggl( \int_{0}^{t}(t-s)^{(\mu -1)/(1- \frac{1}{p})}\,ds \biggr)^{1-\frac{1}{p}}\cdot \biggl( \int_{0}^{t}h^{p}_{r}(s)\,ds \biggr)^{\frac{1}{p}} \\ &\leq \frac{M\Vert u_{0} \Vert }{\Gamma (\nu (1-\mu )+\mu )}+\frac{Mt^{(1- \nu )(1-\mu )}}{\Gamma (\mu )} \bigl(l_{p,\mu }b^{\mu -\frac{1}{p}} \Vert h_{r} \Vert _{L^{p}} \bigr), \end{aligned}$$

where $l_{p,\mu }= (\frac{p-1}{p\mu -1} )^{\frac{p-1}{p}}$.

Dividing both sides of (3.3) by *r* and taking the lower limit as $r\rightarrow +\infty $, by (3.1) we get

$$ 1\leq \frac{M\rho t^{(1-\nu )(1-\mu )}}{\Gamma (\mu )} l_{p,\mu }b ^{\mu -\frac{1}{p}}< 1, $$

which is a contradiction. Therefore $Q(B_{r})\subset B_{r}$ for some $r>0$.

Step 2. Now we show that *Q* is continuous from $B_{r}$ into $B_{r}$. To show this, for any $u, u_{n}\in B_{r}, n=1,2,\ldots $ , with $\lim_{n\rightarrow \infty }\Vert u_{n}-u \Vert _{\nu ,\mu }=0$, we get

$$ \lim_{n\rightarrow \infty }u_{n}(t)=u(t) $$

for all $t\in J$. By the continuous of the nonlinear function *f*, for any $t\in J$ and $0\leq \tau_{k}\leq t$, $k=1,2,\ldots ,m$, we get that

$$ \lim_{n\rightarrow \infty } \bigl\Vert f \bigl(t,u_{n} \bigl( \tau_{1}(t) \bigr),\ldots ,u_{n} \bigl(\tau_{m}(t) \bigr) \bigr)-f \bigl(t,u \bigl(\tau_{1}(t) \bigr),\ldots ,u \bigl( \tau_{m}(t) \bigr) \bigr) \bigr\Vert =0. $$

On the one hand, by assumption (H2) we get that, for all $t\in J,0 \leq s\leq t$, and $0\leq \tau_{k}(s)\leq s$, $k=1,2,\ldots ,m$,

$$\begin{aligned} (t-s)^{\mu -1} \bigl\Vert f \bigl(s,u_{n} \bigl( \tau_{1}(s) \bigr),\ldots ,u_{n} \bigl(\tau_{m}(s) \bigr) \bigr)-f \bigl(s,u \bigl( \tau_{1}(s) \bigr),\ldots ,u \bigl( \tau_{m}(s) \bigr) \bigr) \bigr\Vert \leq 2 (t-s)^{\mu -1} h _{r}(s). \end{aligned}$$

On the other hand, the function $s\rightarrow 2 (t-s)^{\mu -1} h_{r}(s)$ is integrable for $s\in [0,t)$ and $t\in J$. By the Lebesgue dominated convergence theorem we have

$$\begin{aligned}& \int_{0}^{t}(t-s)^{\mu -1} \bigl\Vert f \bigl(s,u_{n} \bigl(\tau_{1}(s) \bigr),\ldots ,u_{n} \bigl(\tau_{m}(s) \bigr) \bigr) \\& \quad {}-f \bigl(s,u \bigl( \tau_{1}(s) \bigr),\ldots ,u \bigl( \tau_{m}(s) \bigr) \bigr) \bigr\Vert \,ds\rightarrow 0\quad \mbox{as }n \rightarrow \infty . \end{aligned}$$

For $t\in J$ and $u_{n},u\in B_{r}$, we have

$$\begin{aligned} & \bigl\Vert t^{(1-\nu )(1-\mu )}(Qu_{n}) (t)-t^{(1-\nu )(1-\mu )}(Qu) (t) \bigr\Vert \\ &\quad \leq \frac{Mt^{(1-\nu )(1-\mu )}}{\Gamma (\mu )} \\ &\quad \quad {}\cdot \int_{0}^{t}(t-s)^{\mu -1} \bigl\Vert f \bigl(s,u_{n} \bigl(\tau_{1}(s) \bigr),\ldots ,u_{n} \bigl(\tau_{m}(s) \bigr) \bigr)- f \bigl(s,u \bigl( \tau_{1}(s) \bigr),\ldots ,u \bigl(\tau_{m}(s) \bigr) \bigr) \bigr\Vert \,ds \\ &\quad \rightarrow 0\quad \mbox{as }n\rightarrow \infty , \end{aligned}$$

which implies that $Qu_{n}\rightarrow Qu$ uniformly on *J* as $n\rightarrow \infty $, and so $Q :B_{r} \rightarrow B_{r}$ is a continuous operator.

Step 3. We will prove that $\{Qu:u\in B_{r}\}$ is an equicontinuous family of functions. For any $u\in B_{r}$ and $0\leq t_{1} < t_{2} \leq b$, by (3.2) and assumptions (H1) and (H2) we get that

$$\begin{aligned} & \bigl\Vert t_{2}^{(1-\nu )(1-\mu )}(Qu) (t_{2})-t_{1}^{(1-\nu )(1-\mu )}(Qu) (t_{1}) \bigr\Vert \\ &\quad \leq \bigl\Vert t_{2}^{(1-\nu )(1-\mu )}S_{\nu ,\mu }(t_{2})-t_{1}^{(1-\nu )(1-\mu )}S_{\nu ,\mu }(t_{1}) \bigr\Vert \Vert u_{0} \Vert \\ &\qquad {}+ \int_{0}^{t_{2}}t_{2}^{(1-\nu )(1-\mu )}K_{\mu }(t_{2}-s)f \bigl(s,u \bigl(\tau_{1}(s) \bigr), \ldots ,u \bigl( \tau_{m}(s) \bigr) \bigr)\,ds \\ &\qquad {}- \int_{0}^{t_{1}}t_{1}^{(1-\nu )(1-\mu )}K_{\mu }(t_{1}-s)f \bigl(s,u \bigl(\tau_{1}(s) \bigr), \ldots ,u \bigl( \tau_{m}(s) \bigr) \bigr)\,ds \\ &\quad \leq \bigl( \bigl\Vert t_{2}^{(1-\nu )(1-\mu )}S_{\nu ,\mu }(t_{2})-t_{2}^{(1-\nu )(1-\mu )}S_{\nu ,\mu }(t_{1}) \bigr\Vert \\ &\quad \quad {}+ \bigl\Vert t_{2}^{(1-\nu )(1-\mu )}S_{\nu ,\mu }(t_{1})-t_{1}^{(1-\nu )(1-\mu )}S_{\nu ,\mu }(t_{1})\bigr\Vert \bigr) \Vert u_{0} \Vert \\ &\qquad {}+ \biggl\Vert \int^{t_{2}}_{t_{1}}t_{2}^{(1-\nu )(1-\mu )}K_{\mu }(t_{2}-s)f \bigl(s,u \bigl(\tau_{1}(s) \bigr),\ldots ,u \bigl( \tau_{m}(s) \bigr) \bigr)\,ds \biggr\Vert \\ &\qquad {}+ \biggl\Vert \int^{t_{1}}_{0}t_{2}^{(1-\nu )(1-\mu )}K_{\mu }(t_{2}-s)f \bigl(s,u \bigl(\tau_{1}(s) \bigr),\ldots ,u \bigl( \tau_{m}(s) \bigr) \bigr)\,ds \\ &\qquad {}- \int^{t_{1}}_{0}t_{1}^{(1-\nu )(1-\mu )}K_{\mu }(t_{2}-s)f \bigl(s,u \bigl(\tau_{1}(s) \bigr), \ldots ,u \bigl( \tau_{m}(s) \bigr) \bigr)\,ds \biggr\Vert \\ &\qquad {}+ \biggl\Vert \int^{t_{1}}_{0}t_{1}^{(1-\nu )(1-\mu )}K_{\mu }(t_{2}-s)f \bigl(s,u \bigl(\tau_{1}(s) \bigr),\ldots ,u \bigl( \tau_{m}(s) \bigr) \bigr)\,ds \\ &\qquad {}- \int^{t_{1}}_{0}t_{1}^{(1-\nu )(1-\mu )}K_{\mu }(t_{1}-s)f \bigl(s,u \bigl(\tau_{1}(s) \bigr), \ldots ,u \bigl( \tau_{m}(s) \bigr) \bigr)\,ds \biggr\Vert \\ &\quad =I_{1}+I_{2}+I_{3}+I_{4}+I_{5}, \end{aligned}$$

where

$$\begin{aligned}& I_{1}= \bigl( \bigl\Vert t_{2}^{(1-\nu )(1-\mu )}S_{\nu ,\mu }(t_{2})-t_{2}^{(1-\nu )(1-\mu )}S_{\nu ,\mu }(t_{1})\bigr\Vert \bigr) \Vert u_{0} \Vert , \\& I_{2}= \bigl( \bigl\Vert t_{2}^{(1-\nu )(1-\mu )}S_{\nu ,\mu }(t_{1})-t_{1}^{(1-\nu )(1-\mu )}S_{\nu ,\mu }(t_{1})\bigr\Vert \bigr) \Vert u_{0} \Vert , \\& I_{3}= \biggl\Vert \int^{t_{2}}_{t_{1}}t_{2}^{(1-\nu )(1-\mu )}K_{\mu }(t_{2}-s)f \bigl(s,u \bigl(\tau_{1}(s) \bigr),\ldots ,u \bigl( \tau_{m}(s) \bigr) \bigr)\,ds \biggr\Vert , \\& I_{4}= \biggl\Vert \int^{t_{1}}_{0}t_{2}^{(1-\nu )(1-\mu )}K_{\mu }(t_{2}-s)f \bigl(s,u \bigl(\tau_{1}(s) \bigr),\ldots ,u \bigl( \tau_{m}(s) \bigr) \bigr)\,ds \\& \hphantom{I_{4}=}- \int^{t_{1}}_{0}t_{1}^{(1-\nu )(1-\mu )}K_{\mu }(t_{2}-s)f \bigl(s,u \bigl(\tau_{1}(s) \bigr),\ldots ,u \bigl( \tau_{m}(s) \bigr) \bigr)\,ds \biggr\Vert , \\& I_{5}= \biggl\Vert \int^{t_{1}}_{0}t_{1}^{(1-\nu )(1-\mu )}K_{\mu }(t_{2}-s)f \bigl(s,u \bigl(\tau_{1}(s) \bigr),\ldots ,u \bigl( \tau_{m}(s) \bigr) \bigr)\,ds \\& \hphantom{I_{5}=}- \int^{t_{1}}_{0}t_{1}^{(1-\nu )(1-\mu )}K_{\mu }(t_{1}-s)f \bigl(s,u \bigl(\tau_{1}(s) \bigr),\ldots ,u \bigl( \tau_{m}(s) \bigr) \bigr)\,ds \biggr\Vert . \end{aligned}$$

Here we calculate

3.4 $$ \big\Vert t_{2}^{(1-\nu )(1-\mu )}(Qu) (t_{2})-t_{1}^{(1-\nu )(1-\mu )}(Qu)(t_{1}) \big\Vert \leq \sum_{i=1}^{5} \Vert I_{i} \Vert . $$

Therefore we have to check that $\Vert I_{i} \Vert $ tend to 0 as $t_{2} \rightarrow t_{1},i=1,2,\ldots ,5$.

For $I_{1}$, by Lemma 2.9 we get

$$\begin{aligned} I_{1}&= \bigl( \bigl\Vert t_{2}^{(1-\nu )(1-\mu )}S_{\nu ,\mu }(t_{2})-t_{2}^{(1-\nu )(1-\mu )}S_{\nu ,\mu }(t_{1}) \bigr\Vert \bigr) \Vert u_{0} \Vert \\ &\leq \bigl\Vert t_{2}^{(1-\nu )(1-\mu )} \bigl(S_{\nu ,\mu }(t_{2})-S_{\nu ,\mu }(t_{1}) \bigr) \bigr\Vert \Vert u_{0} \Vert \rightarrow 0\quad \mbox{as }t_{2} \rightarrow t_{1}. \end{aligned}$$

For $I_{2}$, by Lemma 2.8 we get

$$\begin{aligned} I_{2}&= \bigl( \bigl\Vert t_{2}^{(1-\nu )(1-\mu )}S_{\nu ,\mu }(t_{1})-t_{1}^{(1-\nu )(1-\mu )}S_{\nu ,\mu }(t_{1})\bigr\Vert \bigr) \Vert u_{0} \Vert \\ &\leq \frac{Mb^{(\nu -1)(\mu -1)}}{\Gamma (\nu (1-\mu )+\mu )} \bigl\Vert t_{2}^{(1-\nu )(1-\mu )}-t_{1}^{(1-\nu )(1-\mu )} \bigr\Vert \rightarrow 0\quad \mbox{as } t_{2}\rightarrow t_{1}. \end{aligned}$$

For $I_{3}$, by Lemma 2.8 and (H2) we have

$$\begin{aligned} I_{3} &= \biggl\Vert \int^{t_{2}}_{t_{1}}t_{2}^{(1-\nu )(1-\mu )}K_{\mu }(t_{2}-s)f \bigl(s,u \bigl(\tau_{1}(s) \bigr),\ldots ,u \bigl( \tau_{m}(s) \bigr) \bigr)\,ds \biggr\Vert \\ &\leq \frac{Mt_{2}^{(1-\nu )(1-\mu )}}{\Gamma (\mu )} \int_{0}^{t_{2}}(t _{2}-s)^{\mu -1}h_{r}(s)\,ds \\ &=Mt_{2}^{(1-\nu )(1-\mu )}I^{\mu }_{0+}h_{r}(t_{2}) \rightarrow 0 \quad \mbox{as } t_{2}\rightarrow t_{1}. \end{aligned}$$

For $I_{4}$, by Lemma 2.9 and (H2) we have

$$\begin{aligned} I_{4}&= \biggl\Vert \int^{t_{1}}_{0}t_{2}^{(1-\nu )(1-\mu )}K_{\mu }(t_{2}-s)f \bigl(s,u \bigl(\tau_{1}(s) \bigr),\ldots ,u \bigl( \tau_{m}(s) \bigr) \bigr)\,ds \\ &\quad {}- \int^{t_{1}}_{0}t_{1}^{(1-\nu )(1-\mu )}K_{\mu }(t_{2}-s)f \bigl(s,u \bigl(\tau_{1}(s) \bigr),\ldots ,u \bigl( \tau_{m}(s) \bigr) \bigr)\,ds \biggr\Vert \\ &\leq \frac{2M}{\Gamma (\mu )} \int^{t_{1}}_{0} \bigl[t_{2}^{(1-\nu )(1- \mu )}(t_{2}-s)^{\mu -1}-t_{1}^{(1-\nu )(1-\mu )}(t_{1}-s)^{\mu -1} \bigr]h_{r}(s)\,ds, \end{aligned}$$

and $\int^{t_{1}}_{0}-t_{1}^{(1-\nu )(1-\mu )}(t_{1}-s)^{\mu -1}h_{r}(s)\,ds$ exists. Then by the Lebesgue dominated convergence theorem we have

$$ \int^{t_{1}}_{0} \bigl[t_{2}^{(1-\nu )(1-\mu )}(t_{2}-s)^{\mu -1}-t_{1} ^{(1-\nu )(1-\mu )}(t_{1}-s)^{\mu -1} \bigr]h_{r}(s)\,ds \rightarrow 0\quad \mbox{as } t_{2}\rightarrow t_{1}. $$

For $I_{5}$, by Lemma 2.9 and (H2) we have

$$\begin{aligned} I_{5} &= \biggl\Vert \int^{t_{1}}_{0}t_{1}^{(1-\nu )(1-\mu )}K_{\mu }(t_{2}-s)f \bigl(s,u \bigl(\tau_{1}(s) \bigr),\ldots ,u \bigl( \tau_{m}(s) \bigr) \bigr)\,ds \\ &\quad {}- \int^{t_{1}}_{0}t_{1}^{(1-\nu )(1-\mu )}K_{\mu }(t_{1}-s)f \bigl(s,u \bigl(\tau_{1}(s) \bigr), \ldots ,u \bigl( \tau_{m}(s) \bigr) \bigr)\,ds \biggr\Vert \\ &\leq \biggl\Vert \int^{t_{1}}_{0}t_{1}^{(1-\nu )(1-\mu )} \bigl[K_{\mu }(t_{2}-s)-K_{\mu }(t_{1}-s) \bigr]f \bigl(s,u \bigl(\tau_{1}(s) \bigr),\ldots ,u \bigl( \tau_{m}(s) \bigr) \bigr)\,ds \biggr\Vert \\ &\leq \bigl\Vert K_{\mu }(t_{2}-s)-K_{\mu }(t_{1}-s) \bigr\Vert \int^{t_{1}}_{0}t_{1}^{(1-\nu )(1-\mu )}f \bigl(s,u \bigl(\tau_{1}(s) \bigr),\ldots ,u \bigl( \tau_{m}(s) \bigr) \bigr)\,ds \\ &\rightarrow 0 \quad \mbox{as }t_{2}\rightarrow t_{1}. \end{aligned}$$

In conclusion,

$$ \bigl\Vert t_{2}^{(1-\nu )(1-\mu )}(Qu) (t_{2})-t_{1}^{(1-\nu )(1-\mu )}(Qu) (t_{1}) \bigr\Vert \rightarrow 0 $$

as $t_{2}\rightarrow t_{1}$, which means that the operator $Q:B_{r} \rightarrow B_{r}$ is equicontinuous.

Let $H=\overline{{\operatorname{co}}}Q(B_{r})$. Then it is easy to verify that *Q* maps *H* into itself and $H \subset B_{r}$ is equicontinuous.

Step 4. Now, we prove that $Q : H \rightarrow H$ is a condensing operator. For any $D\subset H$, by Lemma 2.2 there exists a countable set $D_{0} = \{u_{n}\} \subset D$ such that

$$ \alpha \bigl(Q(D) \bigr)\leq 2\alpha \bigl(Q(D_{0}) \bigr). $$

By the equicontinuity of *H* we know that $D_{0} \subset D$ is also equicontinuous.

For $t \in J$, by the definition of *Q* and (H3) we have

$$\begin{aligned} &\alpha \bigl(Q(D_{0}) (t) \bigr) \\ &\quad =\alpha \biggl( \biggl\{ t^{(1-\nu )(1-\mu )}S_{ \nu ,\mu }(t)u_{0} \\ &\quad \quad {}+ \int_{0}^{t}t^{(1-\nu )(1-\mu )}K_{\mu }(t-s)f \bigl(s,u_{n} \bigl(\tau_{1}(s) \bigr), \ldots ,u_{n} \bigl(\tau_{m}(s) \bigr) \bigr)\,ds \biggr\} \biggr) \\ &\quad \leq \frac{2M}{\Gamma (\alpha )}t^{(1-\nu )(1-\mu )} \int_{0}^{t}(t-s)^{ \mu -1}\alpha \bigl( \bigl\{ f \bigl(s,u_{n} \bigl(\tau_{1}(s) \bigr),\ldots ,u_{n} \bigl(\tau_{m}(s) \bigr) \bigr) \bigr\} \bigr)\,ds \\ &\quad \leq \frac{2M}{\Gamma (\alpha )}t^{(1-\nu )(1-\mu )} \int_{0}^{t}(t-s)^{ \mu -1} \bigl[L_{1}\alpha \bigl(D_{0} \bigl(\tau_{1}(s) \bigr) \bigr)+\cdots +L_{m}\alpha \bigl(D _{0} \bigl( \tau_{m}(s) \bigr) \bigr) \bigr]\,ds \\ &\quad \leq \frac{2M}{\Gamma (\mu )}\sum_{k=1}^{m}L_{k}t^{(1-\nu )(1- \mu )} \int_{0}^{t}(t-s)^{\mu -1}\alpha \bigl(D_{0}(s) \bigr)\,ds \\ &\quad \leq \frac{2Mb^{\mu }t^{(1-\nu )(1-\mu )}}{\Gamma (1+\mu )}\sum_{k=1} ^{m}L_{k}\alpha (D). \end{aligned}$$

Since $Q(D_{0}) \subset H$ is bounded and equicontinuous, we know from Lemma 2.3 that

$$ \alpha \bigl(Q(D_{0}) \bigr)=\max_{t\in I}\alpha \bigl(Q(D_{0}) (t) \bigr). $$

Therefore we have

$$ \alpha \bigl(Q(D) \bigr)\leq \frac{4Mb^{\mu }t^{(1-\nu )(1-\mu )}}{\Gamma (1+ \mu )}\sum _{k=1}^{m}L_{k} \alpha (D)\leq \alpha (D). $$

Thus, $Q : B_{r} \rightarrow B_{r}$ is a *k*-set-contractive operator. It follows from Lemma 2.5 that *Q* has at least one fixed point $u \in B_{r}$, which is just a mild solution of problem (1.1) on the interval *J*. □

We further present two special cases.

Case 1. When $B=I$, then $D(B)=E$. We assume that *A*-generate a norm-continuous semigroup $\{W(t)\}_{t\geq 0}$ of uniformly bounded linear operators on *E*. Then from the proof of Theorem 3.1 we have the following theorem.

### Theorem 3.2

*Assume that the nonlinear function* $f:J\times E^{m}\rightarrow E$ *is continuous and the assumptions* (H1)-(H3) *are satisfied*, *then the problem*

$$ \left \{ \textstyle\begin{array}{ll} D^{\nu ,\mu }_{0+}u(t)=Au(t)+f(t,u(\tau_{1}(t)),\ldots ,u(\tau_{m}(t))), \quad t\in J, \\ I^{(1-\nu )(1-\mu )}_{0+}u(0)=u_{0}, \end{array}\displaystyle \right . $$

*has at least one mild solution in* $C^{\nu ,\mu }(J,E)$.

Case 2. When $B=I$ and $\nu =1$, $D(B)=E$. We assume that *A*-generate a norm-continuous semigroup $\{W(t)\}_{t\geq 0}$ of uniformly bounded linear operators on *E*. Then from the proof of Theorem 3.1 we have the following theorem.

### Theorem 3.3

*Assume that the nonlinear function* $f:J\times E^{m}\rightarrow E$ *is continuous and the assumptions* (H1)-(H3) *are satisfied*. *Then the problem*

3.5 $$ \left \{ \textstyle\begin{array}{ll} ^{C}D^{\mu }_{0+}u(t)=Au(t)+f(t,u(\tau_{1}(t)),\ldots ,u(\tau_{m}(t))), \quad t\in J, \\ u(0)=u_{0}, \end{array}\displaystyle \right . $$

*has at least one mild solution in* $C(J,E)$.

### Remark 3.1

For problem (3.5), see [34] for more detail.

## Applications

In this section, we present two examples, which illustrate the applicability of our main results.

### Example 4.1

We consider the following fractional diffusion equations of Sobolev type with delay:

4.1 $$ \textstyle\begin{cases} D^{\nu ,\mu }_{0+} (u(t,x)-\frac{\partial^{2}u(t,x)}{\partial x ^{2}} ) \\ \quad =\frac{\partial^{2}}{\partial x^{2}}u(t,x)+\widetilde{f}(t,u( \tau_{1}(t),x),\ldots ,u(\tau_{m}(t),x)),&x\in \Omega , t\in J, \\ u(t,x)=0,&x\in \partial \Omega ,t\in J, \\ I^{(1-\nu )(1-\mu )}_{0+} (u(0,x)-\frac{\partial^{2}}{\partial x^{2}}u(0,x) )=\widetilde{\varphi }(x),&x\in \Omega , \end{cases} $$

where $D^{\nu ,\mu }_{0+}$ is the Hilfer fractional derivative, $0\leq \nu \leq 1, 0<\mu <1$, $\tau_{k}:J\rightarrow J$ are continuous functions such that $0\leq \tau_{k}(t)< t,k=1,2,\ldots ,m$, $\Omega \subset R^{m}$ is a bounded domain with a sufficiently smooth boundary *∂*Ω, and $\widetilde{f}:J\times R^{m}\rightarrow R$ is continuous.

Let $E = L^{2}(\Omega )$ be the Banach space with the $L^{2}$-norm $\Vert \cdot \Vert _{2}$. We define

$$ D(A)=D(B)=H^{2}(\Omega ),\qquad Au=\frac{\partial^{2}u}{\partial x^{2}}, \qquad Bu=u- \frac{ \partial^{2}u}{\partial x^{2}}, $$

where $H^{2}(\Omega )$ is the completion of the space $C^{2}(\Omega )$ with respect to the norm

$$ \Vert u \Vert _{H^{2}(\Omega )}= \biggl( \int_{\Omega }\sum_{\vert \mu \vert \leq 2} \bigl\vert D^{\mu }u(x) \bigr\vert ^{2}\,dx \biggr)^{\frac{1}{2}}, $$

$C^{2}(\Omega )$ is the set of all continuous functions on *R* that have continuous partial derivatives of order less than or equal to 2. In view of [19], it is easy to see that the pair $(A,B) $ generates a propagation family $W(t)$ of uniformly bounded operators, and similarly to the proof of (2.15), (2.16), and (2.17) in [19], we can see that $\{W(t)\}_{t\geq 0}$ is norm-continuous for $t>0$ and $\Vert W(t) \Vert \leq 1$, that is, assumption (H1) is satisfied.

Let

$$\begin{aligned}& f \bigl(t,u \bigl(\tau_{1}(t),x \bigr),\ldots ,u \bigl( \tau_{m}(t),x \bigr) \bigr)=B^{-1}\widetilde{f} \bigl(t,u \bigl( \tau_{1}(t),x \bigr),\ldots ,u \bigl(\tau_{m}(t),x \bigr) \bigr), \\& \varphi (\cdot )=u_{0}=B^{-1}\widetilde{\varphi }(\cdot ). \end{aligned}$$

Then equation (4.1) can be rewritten in the abstract form as (1.1).

To study this problem, we assume the following conditions:

(i) There exists a essential bounded function $h_{r}(t)$ such that, for any $t\in [0,b]$, $x\in \Omega $, and $u\in L^{2}(\Omega )$ satisfying $(\int_{\Omega }\vert u(x)^{2} \vert \,dx)^{\frac{1}{2}}\leq r$ for some $r>0$, we have

$$ \biggl( \int_{\Omega } \bigl\vert \widetilde{f} \bigl(t,u \bigl( \tau_{1}(t),x \bigr),\ldots ,u \bigl(\tau_{m}(t),x \bigr) \bigr) \bigr\vert ^{2}\,dx \biggr)^{\frac{1}{2}}\leq h_{r}(t). $$

(ii) The function $\widetilde{f}(t,u(\tau_{1}(t),x),\ldots ,u(\tau_{m}(t),x))$ is Lipschitz with respect to variables $u(\tau_{1}(t),x), \ldots ,u(\tau_{m}(t),x)$ with positive constants $l_{k}$ for any $x\in \Omega $ and $k=1,2,\ldots ,m$.

### Theorem 4.1

*If assumptions* (i)-(iii) *are satisfied*, *then problem* (4.1) *has at least one mild solution*, *provided that*

4.2 $$ \frac{Mb^{\mu }t^{(1-\nu )(1-\mu )}}{\Gamma (1+\mu )}\sum_{k=1}^{m}L_{k}< \frac{1}{4}. $$

### Proof

By assumptions (i)-(ii) we can easily verify that conditions (H2)-(H3) are satisfied with $L_{k}=l_{k}$ ($k=1,2,\ldots ,m$). Furthermore, also from assumptions (i)-(ii), combined with assumption (4.2), we know that (3.1) are satisfied. Therefore, Theorem 3.1 follows. □

### Example 4.2

We consider the initial boundary value problem to the following nonlinear time fractional reaction-diffusion equation with delay introduced in [31, 32]:

4.3 $$ \textstyle\begin{cases} D^{\nu ,\mu }_{0+} (u(x,t)-a(t)\Delta u(x,t) )-a(t)\Delta u(x,t) \\ \quad {}= \widetilde{f}(t,u(\tau_{1}(t),x),\ldots ,u(\tau_{m}(t),x)),&x\in \Omega , t\in J, \\ u(t,x)=0,&x\in \partial \Omega ,t\in J, \\ I^{(1-\nu )(1-\mu )}_{0+} (u(0,x)-a(0)\Delta u(0,x) )= \widetilde{\varphi }(x),&x\in \Omega . \end{cases} $$

where $D^{\nu ,\mu }_{0+}$ is the Hilfer fractional derivative, $0\leq \nu \leq 1, 0<\mu <1$, $J=[0,b]$, *m* is a positive integer number, the diffusion coefficient $a(t)$ is continuous on *J* and $\vert a(t_{2})-a(t_{1}) \vert \leq C\vert t_{2}-t_{1} \vert ^{\gamma }, 0<\gamma \leq 1, t _{1},t_{2}\in J$, *C* is a positive constant independent of $t_{1}$ and $t_{2}$, Δ is the Laplace operator, $\tau_{k}:J \rightarrow J$ are continuous function such that $0\leq \tau_{k}(t)< t,k=1,2, \ldots ,m$, $\Omega \subset R^{m}$ is a bounded domain with a sufficiently smooth boundary *∂*Ω, $\widetilde{f}:J \times R^{m}\rightarrow R$ is continuous, and $\varphi \in L^{2}( \Omega )$.

Let $E = L^{2}(\Omega )$ be the Banach space with the $L^{2}$-norm $\Vert \cdot \Vert _{2}$. We define

$$ D(A)=D(B)=H^{2}(\Omega )\cap H_{0}^{1}(\Omega ),\qquad Au=a(t)\Delta u,\qquad Bu=u-a(t) \Delta u, $$

where $H^{2}(\Omega )$ is the completion of the space $C^{2}(\Omega )$ with respect to the norm

$$ \Vert u \Vert _{H^{2}(\Omega )}= \biggl( \int_{\Omega }\sum_{\vert \mu \vert \leq 2} \bigl\vert D^{\mu }u(x) \bigr\vert ^{2}\,dx \biggr)^{\frac{1}{2}}. $$

($C^{2}(\Omega )$ is the set of all continuous functions on Ω that have continuous partial derivatives of order less than or equal to 2.) $H_{0}^{1}(\Omega )$ is the completion of $C^{1}( \Omega )$ with respect to the norm $\Vert u \Vert _{H^{1}(\Omega )}$, and $C_{0}^{1}(\Omega )$ is the set of all functions $u\in C^{1}(\Omega )$ with compact supports on the domain Ω. In view of [19], it is easy to see that the pair $(A,B) $ generates a propagation family $W(t)$ of uniformly bounded, and similarly to the proof of (2.15), (2.16), and (2.17) in [19], we can see that $\{W(t)\}_{t\geq 0}$ is norm-continuous for $t>0$ and $\Vert W(t) \Vert \leq 1$, that is, assumption (H1) is satisfied.

Let

$$\begin{aligned}& u(t) = u(\cdot , t), \\& f \bigl(t, u \bigl(\tau_{1}(t) \bigr), \ldots , u \bigl(\tau_{m}(t) \bigr) \bigr) ( \cdot ) = g \bigl(t, u \bigl( \cdot , \tau_{1}(t) \bigr), \ldots , u \bigl(\cdot , \tau_{m}(t) \bigr) \bigr) \\& \hphantom{ f \bigl(t, u \bigl(\tau_{1}(t) \bigr), \ldots , u \bigl(\tau_{m}(t) \bigr) \bigr) ( \cdot ) }=B^{-1}\widetilde{f} \bigl(t,u \bigl(\tau_{1}(t),x \bigr), \ldots ,u \bigl(\tau_{m}(t),x \bigr) \bigr), \\& \varphi (\cdot )= u_{0} -a(0)\Delta u_{0}= B^{-1}\widetilde{\varphi }( \cdot ). \end{aligned}$$

Then the initial boundary value problem of the nonlinear time fractional reaction-diffusion equation with delay (4.3) can be transformed into the abstract form of problem (1.1).

### Theorem 4.2

*Suppose that the following assumptions are satisfied*:

(i) *There exists an essentially bounded function* $h_{r}(t)$ *such that*, *for any* $t\in [0,b],x\in \Omega $, *and* $u\in L^{2}(\Omega )$ *satisfying* $(\int_{\Omega }\vert u(x)^{2} \vert \,dx)^{\frac{1}{2}}\leq r$ *for some* $r>0$,
  $$ \biggl( \int_{\Omega } \bigl\vert \widetilde{f} \bigl(t,u \bigl( \tau_{1}(t),x \bigr),\ldots ,u \bigl(\tau_{m}(t),x \bigr) \bigr) \bigr\vert ^{2}\,dx \biggr)^{\frac{1}{2}}\leq h_{r}(t); $$
(ii) *The function* $\widetilde{f}(t,u(\tau_{1}(t),x),\ldots ,u(\tau_{m}(t),x))$ *is Lipschitz in variables* $u(\tau_{1}(t),x),\ldots ,u(\tau_{m}(t),x)$ *with positive constants* $l_{k}$ *for any* $x\in \Omega$ *and* $k=1,2,\ldots ,m$.

*Then problem* (4.2) *has at least one mild solution*, *provided that*

4.4 $$ \frac{Mb^{\mu }t^{(1-\nu )(1-\mu )}}{\Gamma (1+\mu )}\sum_{k=1}^{m}L _{k}< \frac{1}{4}. $$

### Proof

By assumptions (i)-(ii) we can easily verify that conditions (H2)-(H3) are satisfied with $L_{k}=l_{k}$ ($k=1,2,\ldots ,m$). Furthermore, also from assumptions (i)-(ii) combined with assumption (4.2) we know that (3.1) are satisfied. Therefore, our Theorem 3.1 follows. □

## Conclusions

In this paper, we deal with a class of nonlinear fractional nonautonomous evolution equations with delay by using the Hilfer fractional derivative, which generalizes the famous Riemann-Liouville fractional derivative. The definition of mild solutions for the studied problem was given based on an operator family generated by the operator pair $(A,B)$ and probability density function. Combining the techniques of fractional calculus, measure of noncompactness, and fixed point theorem with respect to a *k*-set-contractive operator, we obtain a new result on the existence of mild solutions with the assumption that the nonlinear term satisfies some growth condition and noncompactness measure condition. The results obtained improve and extend some related conclusions on this topic. When $\nu =1$, the fractional equation (1.1) simplifies to a classical Caputo fractional differential equation of Sobolev type with nonlocal conditions studied by Li et al. [35]. When $B=I$, $D(B)=E$. We assume that *A*-generate a norm-continuous semigroup $\{W(t)\}_{t\geq 0}$ of uniformly bounded linear operators on *E*. Then the fractional equation (1.1) simplifies to evolution equation with Hilfer fractional derivative studied by Gu et al. [18].
